# Changes in serological, inflammatory, and serum protein electrophoretic markers during ongoing meglumine antimoniate plus allopurinol therapy in canine leishmaniosis

**DOI:** 10.1007/s11259-026-11275-4

**Published:** 2026-05-16

**Authors:** Jacobo Giner, Ana González, María Eugenia Lebrero, Pablo Quilez, David Guallar, Álex Gómez, Diana Marteles-Aragüés, Sergio Villanueva-Saz

**Affiliations:** 1 Menescalia Veterinary Clinic, Ismael Merlo Actor, Valencia, Spain; 2 Department of Animal Pathology, Veterinary Faculty, University of Zaragoza, Calle Miguel Servet, 177, Zaragoza, 50013 Spain; 3 Clinical Immunology Laboratory, Veterinary Faculty, University of Zaragoza, Calle Miguel Servet, 177, Zaragoza, 50013 Spain; 4 Instituto Agroalimentario de Aragón-IA2 (Universidad de Zaragoza-CITA), Calle Miguel Servet, 177, Zaragoza, 50013 Spain

**Keywords:** Circulating immune complexes, Dog, Follow-up, *Leishmania infantum*, Serology, Serum protein electrophoresis, Treatment

## Abstract

Canine leishmaniosis, caused by *Leishmania infantum*, is a vector-borne disease in which early treatment monitoring remains challenging. This prospective study evaluated short-term changes in serological, inflammatory, and serum protein electrophoretic parameters during first-line therapy with meglumine antimoniate plus allopurinol. Eighteen dogs with confirmed clinical disease were enrolled at diagnosis, and fifteen dogs completing all follow-up visits (Days 0, 7, 14, 21, and 29) were included. Infection was confirmed by compatible clinical and/or laboratory abnormalities, high anti-*Leishmania* antibody levels, and successful parasite isolation. Anti-*Leishmania* antibodies were quantified using in-house single-dilution and two-fold serial dilution ELISA; circulating immune complexes, C-reactive protein, a complete blood count, a biochemical profile, and serum protein electrophoresis were also assessed. Both ELISA methods showed a significant decline in antibody levels during treatment, with serial dilution ELISA showing greater discrimination among highly positive samples. C-reactive protein concentrations decreased significantly from diagnosis to Day 29, indicating reduced systemic inflammation. Serum protein electrophoresis showed significant increases in albumin and the albumin:globulin ratio, along with a significant decrease in gamma globulins. Circulating immune complexes concentrations remained stable. These findings support quantitative serology, C-reactive protein, and serum protein electrophoresis as useful tools for early monitoring of therapeutic response in clinical canine leishmaniosis.

## Introduction

Canine leishmaniosis (CanL) caused by *Leishmania infantum* and transmitted by phlebotomine sand flies, is a zoonotic vector-borne disease of major veterinary and public health relevance in European Mediterranean endemic regions (Plesko et al. 2025). Dogs are widely recognized as the principal domestic reservoir host for zoonotic visceral leishmaniasis due to *L. infantum*, sustaining parasite circulation in peri-domestic settings and facilitating human exposure where competent vectors are present (Alvar et al. 2004). Within a One Health approach, the control and clinical management of CanL are integrally linked to broader prevention strategies, as decreasing canine infectiousness (Miró et al. 2011) and enhancing case detection can reduce transmission pressure in affected communities (Courtenay et al. 2019).

The clinical outcome of *L. infantum* infection in dogs is largely determined by the dog’s immune response to the parasite which may be influenced by the genetic background of the dog (Pinelli et al. 1994). Clinical manifestations may range from absent to mild, typically reflecting a protective immune response dominated by cell-mediated Th1 immunity, characterized by cytokines such as TNF-α and IFN-γ that drive macrophage activation and intracellular parasite killing. Conversely, susceptibility and disease progression are often associated with inadequate cell-mediated control and a predominantly humoral immune milieu, in which high antibody production co-occurs with immunoregulatory cytokines that can attenuate parasite-specific cellular responses (Hosein et al. 2017).

Importantly, in sick dogs, higher anti-*Leishmania* antibody concentrations are associated with greater disease severity and higher clinical scores, particularly in advanced clinical stages (Proverbio et al. 2014). Excessive anti-*Leishmania* antibody production, together with circulating parasite antigens, favors the generation of circulating immune complexes (CICs) that contributes to inflammation and organ damage, particularly renal involvement, a key determinant of prognosis (Parody et al. 2019).

The most frequent clinical manifestations of CanL include peripheral lymphadenomegaly and cutaneous and ocular lesions. In sick dogs, non-specific systemic signs are common and may include weight loss, anorexia, epistaxis, polyarthritis, and renal involvement ranging from proteinuria to chronic kidney disease (Solano-Gallego et al. 2011). Laboratory abnormalities in dogs with leishmaniosis are typically non-specific and may include normocytic, normochromic, non-regenerative anemia, renal azotemia, proteinuria, hyperproteinemia with hypoalbuminemia; and a decreased or inverted albumin-to-globulin ratio. Additional biochemical changes may also occur, depending on the organs and tissues involved (Paltrinieri et al. 2016). In some circumstances, however, atypical clinical signs may be observed (Peris et al. 2022), and recent reports describe clinical cases with an absence of a detectable *Leishmania infantum*–specific humoral response (Villanueva-Saz et al. 2025a).

Several confirmatory techniques are available for the diagnosis of *L. infantum* infection in dogs, including direct methods such as molecular assays, cytology, histology often combined with specific immunohistochemistry, and parasite culture. In contrast, indirect methods, primarily serological tests, include the immunofluorescent antibody test (IFAT), direct agglutination test (DAT), enzyme-linked immunosorbent assay (ELISA), and Western blot (WB) (Maia and Campino 2018). In routine clinical practice, quantitative serological assays such as IFAT or ELISA are most commonly used to confirm infection in symptomatic dogs and to support clinical staging and treatment monitoring. (Solano-Gallego et al. 2011).

Other diagnostic approaches that enable direct demonstration of *Leishmania* spp. include parasite isolation by culture (Maia and Campino 2008), which offers high specificity and is particularly useful for confirming infection in complex cases (Villanueva-Saz et al. 2025b; Villora et al. 2025) and for establishing an association between infection and the clinical presentation in suspected animals. In this context, parasite culture is considered highly specific because the growth of viable promastigotes can be attributed to *Leishmania* spp. when appropriate sampling, including lymph node aspirates and bone marrow aspirates, and proper specimen handling are ensured.

Two main therapeutic regimens are commonly used for the management of clinical canine leishmaniosis: meglumine antimoniate combined with allopurinol and miltefosine combined with allopurinol (Solano-Gallego et al. 2011). Among these, meglumine antimoniate plus allopurinol is generally regarded as a first-line option (Miró et al. 2008). Nevertheless, although most treated dogs achieve clinical remission, they frequently remain persistently infected and may continue to harbor the parasite (Manna et al. 2008). With respect to treatment monitoring, current recommendations do not routinely support the use of serology within the first six months after initiating anti-*Leishmania* therapy, given the historically weak correlation between antibody concentrations and clinical improvement (Solano-Gallego et al. 2011). However, recent evidence indicates that a measurable decline in anti-*Leishmania* antibody levels may be detectable as early as 30 days after the first administration of meglumine antimoniate (Solano-Gallego et al. 2016).

The aims of this study were to evaluate the short-term kinetics of *Leishmania infantum*–specific antibodies in dogs with clinical canine leishmaniosis (CLWG stage C) during first-line treatment with meglumine antimoniate plus allopurinol, using an in-house single-dilution ELISA and a two-fold serial dilution ELISA. In addition, we aimed to assess the temporal evolution of circulating immune complexes and C-reactive protein, and to characterize longitudinal changes in serum protein electrophoretic fractions during the first month of therapy.

## Materials and methods

### Dogs

Eighteen dogs diagnosed with clinical leishmaniosis were enrolled between January 2023 and September 2024 at the time of diagnosis. The diagnosis of CanL was established based on the presence of compatible clinical signs and/or laboratory abnormalities, together with high anti-*Leishmania* antibody titers and successful in vitro parasite isolation. Dogs were classified at the time of diagnosis as previously described by Canine Leishmaniosis Working Group (CLWG) (Oliva et al. 2010). Dogs were treated with meglumine antimoniate administered subcutaneously at a total daily dose of 100 mg/kg, divided into two doses, for 28 days, together with oral allopurinol at 10 mg/kg twice daily for 12 months. The dogs were followed up at days 7 (*n* = 18), 14 (*n* = 17), 21 (*n* = 16) and 29 (*n* = 15) during treatment.

For each animal, five millilitres of blood were collected via jugular venipuncture and divided into two tubes, one tube (1 ml) with EDTA for a Complete Blood Count (CBC) and another one (4 ml) without anticoagulant. Serum samples were used for the detection of specific pathogens’ antibodies, clinical biochemistry, serum protein electrophoresis, and detection of circulating immune complexes. CBC and clinical biochemistry were performed within the first hour after blood collection. Tubes without anticoagulant were maintained at room temperature to obtain serum aliquots, which were stored at -20 °C until processing for the detection of antibodies against specific pathogens, serum protein electrophoresis, and detection of circulating immune complexes.

### Leishmania infantum in vitro isolation

For each dog, parasite isolation was performed using in-house Novy–MacNeal–Nicolle (NNN) medium and commercial Schneider’s medium, both supplemented with 100 IU/mL penicillin, 100 µg/mL streptomycin, and 10% fetal calf serum (Thermo Fisher Scientific). Several drops of aspirated lymph node material were inoculated into the liquid phase of the NNN medium and then incubated in Schneider’s medium at 26 ± 1 °C. Cultures were examined microscopically on a daily basis (Giner et al. 2020). Samples were considered negative when no promastigotes were detected after 5 weeks of incubation.

### Detection of anti-Leishmania antibodies by an in-house single-dilution ELISA

To detect *L. infantum*-specific antibodies, an in-house ELISA (99.37% sensitivity and 97.50% specificity) (Basurco et al. 2020), based on *L. infantum* antigen (MHOM/FR/78/LEM75 zymodeme MON-1) was performed. Briefly, 100 µL of serum, diluted 1:800 in phosphate-buffered saline (PBS) with 0.05% Tween 20 (PBST) and 1% skimmed milk (PBST-M), was added to each well, and plates were incubated for 1 h at 37 °C in a humid chamber. After washing twice with PBS, 100 µL of horseradish peroxidase-conjugated protein A, diluted 1:20,000 in PBST-M, was added. Plates were incubated again for 1 h at 37 °C, washed with PBST and PBS, and incubated with ortho-phenylenediamine and a stable peroxide substrate buffer for 20 ± 5 min at room temperature in the dark. The reaction was stopped by adding 2.5 M H₂SO₄. Absorbance was read at 492 nm with an automatic ELISA reader (Multiskan ELISA reader, Labsystems, Midland, Canada). Each plate included serum samples from a dog infected with *L. infantum* as confirmed by cytological examination as a positive control (calibrator) and serum samples from a healthy, noninfected dog from the blood donor program as a negative control. To avoid ELISA intra-assay variability, all samples from the same dog were analyzed on the same ELISA plate; to minimize inter-assay variability, all ELISA plates were performed on the same day. The cut-off value of ELISA was set to 30 EU (mean + 4 standard deviations of values from 70 healthy dogs from a non-endemic area). The results were quantified as ELISA units (EU) compared to a positive control serum sample used as a calibrator that was arbitrarily set to 100 EU.

### Detection of anti-Leishmania antibodies by an in-house two-fold serial dilution ELISA

All samples with an optical density (OD) equal or higher than 3 were studied using a two-fold serial dilution (Solano-Gallego et al. 2016). The samples were started at 1:800 dilution and continued for 10 further dilutions in the same ELISA plate. To avoid ELISA intra-assay variability, all samples from the same dog were analyzed on the same ELISA plate; to minimize inter-assay variability, all ELISA plates were performed on the same day. The results were quantified as EU related to a calibrator arbitrarily set at 100 EU with an OD of 1.00 at the 1:800 dilution. The values of the dilutions at which the OD was close to 1.00 was chosen for the calculation of the EU using the following formula: (sample OD/calibrator OD) × 100 × dilution factor.

### Detection of CIC by ELISA

Serum concentrations of circulating immune complexes (CICs; mg/L) were measured using a commercial ELISA kit, according to the manufacturer’s instructions (Canine CIC ELISA Kit, BlueGene Biotech, catalogue number E08C0241; sensitivity, 0.1 mg/L; detection range, 2.5–50 mg/L). The kit consists of a competitive enzyme immunoassay technique utilizing a polyclonal anti-CIC antibody and a CIC-horseradish Peroxidase (HRP) conjugate. The assay sample and buffer were incubated together with the CIC-HRP conjugate in precoated plates for one hour. After the incubation period, the wells were decanted and washed five times. The wells were then incubated with a substrate for the HRP enzyme. The product of the enzyme–substrate reaction forms a blue coloured complex. Finally, a stop solution was added to stop the reaction, after which the solution becomes yellow. The intensity of the colour was measured spectrophotometrically at 450 nm using a microplate reader. The intensity of the colour is inversely proportional to the CIC concentration from serum samples, and the CIC-HRP conjugate competes for the anti-CIC antibody binding site.

### Complete blood count (CBC) and clinical biochemistry

For each patient a CBC (Idexx Procyte Dx) and a biochemical profile including glucose, blood urea nitrogen (BUN), creatinine (CREA), calcium (Ca), inorganic phosphorus (P), alanine aminotransferase (ALT), aspartate aminotransferase (AST), alkaline phosphatase (ALP), gamma glutamyl transferase (GGT), total bilirubin (TBIL), amylase (AMY) and C-Reactive Protein (CRP) (Analyzer AmiShield) was performed during the follow-up. In the case of urinalysis with urinary protein creatinine ratio (UPC) (Idexx Catalyst One), was performed at the time of diagnosis.

### Serum protein agarose gel electrophoresis

Serum protein electrophoresis was performed by agarose gel electrophoresis (AGE) (Hydragel PROTEINAS K20 Beta1– Beta2, Sebia, Evry, France). Serum was electrophoresed for 21 min at 92 V and stained with diluted amidoschwarz dye at pH 2 (4 g/L amidoschwarz dye and 6.7% ethylene glycol). The AGE procedure was conducted according to the manufacturer’s instructions and commercial human serum was used as a control (normal control serum; Sebia, Evry, France). The electrophoretic curve for each sample was displayed and read with a GELSCAN TM densitometry system (Sebia, France). The electrophoretic curve for each sample was assessed using the Phoresis software. Protein fractions were determined as a percentage of optical absorbance, and the absolute concentration g/L was automatically calculated from the total serum protein concentration using a spectrophotometer.

### Detection of concurrent vector-borne diseases by commercial immunochromatographic rapid test

The rapid test (Uranotest^®^ Quattro, Urano Diagnostics) was performed for the qualitative detection of *Dirofilaria immitis*–specific antigens and for the qualitative detection of antibodies against *L. infantum*, *Ehrlichia canis*, and *Anaplasma platys*, following the instructions of the manufacturer. All tests were stored at room temperature and were performed as described in the instructions supplied with the test kit.

### Statistical analysis

All statistical analyses were performed using SPSS Statistics 29.0 software (IBM Corp., Chicago, USA). Normal distribution was determined using the Shapiro-Wilk test. Only dogs that completed the full follow-up at the scheduled time points (day 7, day 14, day 21, and day 29) were included in the statistical analysis. The Friedman test was used to assess statistically significant differences between samples. A p value of < 0.05 was considered statistically significant.

## Results

### Animals characterization

Eighteen dogs with confirmed clinical leishmaniosis were enrolled at diagnosis. Of these, fifteen completed all follow-up visits (Days 0, 7, 14, 21, and 29) and were included in the study, whereas the remaining three dogs were lost to follow-up at different time points: Day 7 (*n* = 1) and Day 14 (*n* = 2). At diagnosis, all were classified as stage C (clinically sick) according to the CLWG classification. Among the dogs that completed follow-up, nine were males and six were females; all were sexually intact. Regarding breed, eight dogs were purebred (French Bulldog, Cane Corso, Dachshund, American Staffordshire Terrier, Bull Terrier, German Shepherd, Boxer, and Poodle; *n* = 1 each), whereas the remaining seven were mixed-breed. The median age at diagnosis was 5 years (range, 12 months to 13 years).

### Leishmania infantum in vitro isolation

*Leishmania* spp. was isolated from lymph node material in all dogs included in the study. However, differences in time to culture positivity were observed among isolates. Six of the fifteen isolates were culture-positive after 4 days of incubation, two isolates after 7 days, five isolates after 10 days, and the remaining two isolates after 12 days of incubation in NNN medium. All *Leishmania* spp. strains isolated were molecularly type as isolated as *L. infantum* based on MatMet approach, which involved partial sequencing of the mitochondrial cytB gene using the MinION nanopore platform. The objective of this methodology was to identify the *Leishmania* species present in various organs that had previously tested positive by species-specific qPCR, through partial sequencing of the kinetoplast cytB gene (Villanueva-Saz et al. 2025b).

### Serology

**Fig. 1:**
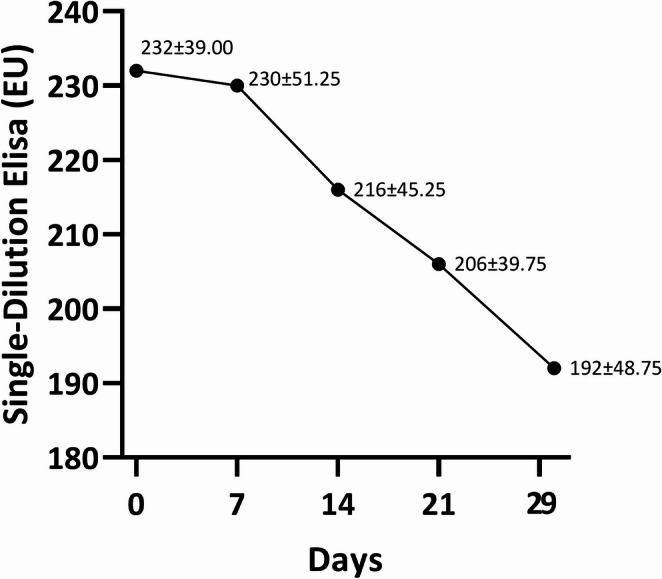
Anti-*Leishmania infantum* antibody levels, expressed as median (interquartile range), at diagnosis (Day 0) and during anti-*Leishmania* treatment (Days 7, 14, 21, and 29) in 15 dogs with clinical leishmaniosis classified as stage C (clinically sick) according to the CLWG classification, as measured by an in-house single-dilution ELISA

For serology, both antibody-based assays showed a clear decreasing pattern over time (Table 1). Single-Dilution ELISA values declined progressively, with median ± interquartile range (IQR) values of day 0 (232; 39.00), day 7 (230; 51.25), day 14 (216; 45.25), day 21 (206; 39.75), and day 29 (192; 48.75) (Fig. 1). The IQRs were relatively narrow in relation to the medians (approximately 17–25%), indicating low inter-individual variability and suggesting that most dogs exhibited comparable antibody levels at each sampling point. This temporal decrease was statistically significant (Friedman test, *p* < 0.001), with significant reductions observed between Day 0 and Day 21 (*p* = 0.005), Day 0 and Day 29 (*p* < 0.001), and between day 7 and subsequent time points (day 21, *p* = 0.009; day 29, *p* < 0.001).

**Table 1:** Laboratorial alterations at the time of diagnosis and during treatment. The median values ± interquartile range of laboratorial parameters at every point of the study.

Parameters (unit)	Reference intervals	Day 0	Day 7	Day 14	Day 21	Day 29	Median across all time points
*Clinical Biochemistry*							
Glucose (mg/dl)	60–110	74.0 ± 12.25	80.5 ± 16.0	77.5 ± 12.75	77.0 ± 15.75	78.0 ± 20.75	77.5 ± 19.25
BUN (mg/dl)	6–36	13.0 ± 18.0	16.0 ± 5.5	15.0 ± 11.0	16.0 ± 8.5	14.5 ± 11.0	15.5 ± 11.5
CREA (mg/dl)	0.8–1.6	0.85 ± 0.33	0.80 ± 0.33	0.80 ± 0.28	0.85 ± 0.28	0.9 ± 0.10	0.80 ± 0.3
Ca (mg/dl)	8.5–11.6	10.30 ± 1.20	10.15 ± 1.75	10.20 ± 1.08	10.00 ± 1.25	10.45 ± 1.70	10.20 ± 1.55
P (mg/dl)	3.2–8.7	5.30 ± 0.68	5.95 ± 1.05	5.85 ± 0.85	5.45 ± 1.00	5.80 ± 1.25	5.70 ± 1.18
ALT (U/l)	10–85	23.0 ± 11.50	27.5 ± 6.25	26.5 ± 17.25	28.0 ± 8.75	30.0 ± 18.75	27.0 ± 12.50
AST (U/l)	10–85	23.0 ± 9.0	21.5 ± 7.50	24.0 ± 11.00	24.5 ± 9.25	26.0 ± 28.75	24.0 ± 10.75
ALP (U/l)	0-110	27.0 ± 6.5	29.0 ± 5.25	28.5 ± 18.5	26.5 ± 10.0	33.0 ± 17.0	28.0 ± 10.8
GGT (U/l)	1–10	1.0 ± 0.0	1.0 ± 0.0	1.0 ± 0.0	1.0 ± 0.0	1.0 ± 0.0	1.0 ± 0.0
TBIL (mg/dl)	0.0-0.2	0.4 ± 0.0	0.3 ± 0.08	0.4 ± 0.15	0.35 ± 0.10	0.3 ± 0.18	0.4 ± 0.1
CRP (µg/ml)	5.0–10.0	34.0 ± 9.0	35.9 ± 27.2	36.5 ± 19.3	26.6 ± 11.5	23.6 ± 11.5	30 ± 14.8
UPC	< 0.5	0.2 ± 0.7	NA	NA	NA	NA	NA
*CBC*							
Red blood cells (M/µl)	5.65–8.87	5.41 ± 1.85	5.26 ± 1.44	5.58 ± 1.32	5.77 ± 0.72	6.25 ± 0.55	5.79 ± 1.44
Hematocrit (%)	37.3–61.7	32 ± 15.3	32.8 ± 13.6	35.5 ± 9.80	35.5 ± 5.58	40 ± 5.62	36.1 ± 12.20
Hemoglobin (g/dl)	13.1–20.5	12.4 ± 4.55	11.8 ± 4.05	11.8 ± 4.03	12.4 ± 1.55	13.8 ± 2.12	12.8 ± 4.10
MCV (fl.)	61.6–73.5	62.9 ± 5.88	65.5 ± 6.60	63.8 ± 8.1	65.2 ± 3.95	64.8 ± 4.72	64.5 ± 6.80
MCH (pg)	21.2–25.9	22.5 ± 1.73	22.6 ± 1.38	22.8 ± 1.55	22.7 ± 0.5	22.7 ± 1.23	22.6 ± 1.62
MCHC (g/dl)	32.0-37.9	35.2 ± 2.32	35.4 ± 1.3	34.5 ± 2.38	34.7 ± 1.58	34.5 ± 1.85	35 ± 1.98
RDW (%)	13.6–21.7	17.6 ± 1.87	17.3 ± 2.07	16.6 ± 2.95	16.7 ± 3.62	16.7 ± 4.65	17.1 ± 3.47
% Reticulocytes		0.8 ± 1.10	0.9 ± 0.38	0.95 ± 1.3	1.5 ± 1.3	0.9 ± 1.6	0.95 ± 1.5
Reticulocytes (K/µl)	10.0-110.0	49.9 ± 71.0	52.9 ± 42.0	53.7 ± 48.4	87.9 ± 75.0	56.5 ± 89.5	60.2 ± 75.1
Reticulocytes haemoglobin (pg)	22.3–29.6	23.1 ± 1.92	23.5 ± 2.05	23.8 ± 4.53	24.1 ± 1.88	23.7 ± 2.10	23.5 ± 2.38
Leucocytes (K/µl)	5.05–16.76	8.69 ± 4.18	10.8 ± 7.92	11.9 ± 3.61	10.9 ± 3.77	10.2 ± 2.20	10.5 ± 4.6
Neutrophils (K/µl)	2.95–11.64	5.44 ± 4.77	7.19 ± 6.36	8.02 ± 2.87	7.02 ± 3.07	6.23 ± 2.33	6.44 ± 4.38
Lymphocytes(K/µl)	1.05–5.10	1.96 ± 0.62	1.73 ± 0.93	2.31 ± 1.77	2.23 ± 1.70	2.43 ± 1.74	2.02 ± 1.56
Monocytes (K/µl)	0.16–1.12	0.67 ± 0.44	0.88 ± 0.69	0.86 ± 0.35	0.79 ± 0.33	0.67 ± 0.23	0.75 ± 0.39
Eosinophils (K/µl)	0.06–1.23	0.25 ± 0.1	0.26 ± 0.22	0.39 ± 0.19	0.42 ± 0.35	0.39 ± 0.20	0.35 ± 0.27
Basophils (K/µl)	0.00-0.10	0.03 ± 0.04	0.04 ± 0.06	0.04 ± 0.05	0.04 ± 0.04	0.02 ± 0.03	0.03 ± 0.05
Platelets (K/µl)	148–484	226.0 ± 118.0	206.0 ± 115.0	201.0 ± 131.0	216.0 ± 54.0	194.0 ± 150.0	210.0 ± 127.0
MVP (fl.)	8.7–13.2	13.4 ± 1.27	13.2 ± 1.95	12.8 ± 1.43	13.7 ± 2.05	13.0 ± 2.35	13.2 ± 2.15
PDW (fl.)	9.1–19.4	13.8 ± 2.10	12.9 ± 1.10	14.2 ± 6.10	14.6 ± 3.55	12.30 ± 1.30	13.6 ± 3.87
*Serum Protein Electrophoresis*							
TP (g/dl)	5.4–7.1	7.30 ± 0.70	7.45 ± 1.78	7.30 ± 1.68	6.95 ± 2.20	7.30 ± 2.90	7.30 ± 2.05
Albumin (%)	47.0–59.0	26.60 ± 12.00	29.10 ± 10.80	34.20 ± 7.12	37.00 ± 10.40	37.70 ± 9.75	32.8 ± 12.1
Albumin (g/dl)	2.6–3.3	1.90 ± 1.10	2.20 ± 0.88	2.40 ± 0.75	2.65 ± 0.63	2.40 ± 0.68	2.40 ± 0.90
Alfa 1 (%)	4.0–7.0	3.25 ± 0.33	3.45 ± 1.10	3.45 ± 0.53	3.60 ± 0.68	3.60 ± 0.85	3.40 ± 0.80
Alfa 1 (g/dl)	0.2–0.5	0.20 ± 0.10	0.25 ± 0.10	0.20 ± 0.10	0.20 ± 0.10	0.20 ± 0.10	0.20 ± 0.10
Alfa 2 (%)	5.0–12.0	13.80 ± 5.75	16.70 ± 8.05	15.10 ± 8.18	15.8 ± 4.90	18.00 ± 6.85	16.20 ± 6.18
Alfa 2 (g/dl)	0.3–1.1	1.05 ± 0.28	1.20 ± 0.35	1.15 ± 0.35	1.15 ± 0.38	1.30 ± 0.40	1.20 ± 0.40
Beta (%)	21.0–38.0	24.10 ± 14.60	22.40 ± 3.20	22.90 ± 5.55	22.40 ± 4.90	21.80 ± 7.13	22.50 ± 7.40
Beta (g/dl)	0.9–1.6	1.85 ± 1.50	1.70 ± 0.75	1.75 ± 0.73	1.70 ± 0.6	1.55 ± 0.8	1.70 ± 0.7
Gamma (%)	8.0–18.0	21.50 ± 22.50	22.00 ± 10.40	18.60 ± 11.70	15.20 ± 12.60	15.20 ± 8.95	18.30 ± 14.60
Gamma (g/dl)	0.3–0.8	1.70 ± 1.53	1.70 ± 0.95	1.45 ± 0.88	1.10 ± 0.63	0.95 ± 0.73	1.40 ± 0.98
Albumin: Globulin		0.37 ± 0.22	0.42 ± 0.23	0.53 ± 0.16	0.59 ± 0.24	0.61 ± 0.25	0.49 ± 0.27
*Serology*							
Single-Dilution Elisa (EU)	> 30	232 ± 39.00	230 ± 51.25	216 ± 45.25	206 ± 39.75	192 ± 48.75	212 ± 53.80
Two-fold serial dilution (EU)	> 30	344,044 ± 170,602	359,103 ± 199,927	325,689 ± 165,035	282,124 ± 161,585	239,619 ± 150,714	318,999 ± 183,111
CIC (mg/l)		18.40 ± 15.70	19.30 ± 14.90	17.90 ± 14.9	17.50 ± 15.00	18.80 ± 14.00	19.20 ± 15.70

Abbreviations:* ALP* alkaline phosphatase, *ALT* alanine aminotransferase, *AST* aspartate aminotransferase, *BUN* blood urea nitrogen, *Ca* Calcium, *CIC* circulating immune complexes, *CREA* creatinine, *CRP* C-Reactive Protein, *GGT* gamma-glutamyl transferase, *MCH* mean corpuscular haemoglobin, *MCHC* mean corpuscular haemoglobin concentration, *MCV* mean corpuscular volume, *MVP* Mean Platelet Volume, *NA* not available, *P* inorganic phosphorus, *PDW* Platelet Distribution Width, *RBC* red blood count, *RDW* red blood cell distribution, *TBIL* total bilirubin, *TP* total proteins, *UPC* urinary protein creatinine ratio

**Fig. 2:**
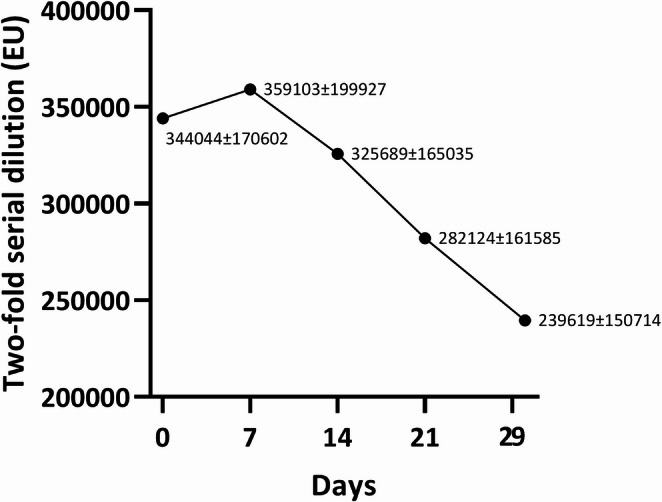
Anti-*Leishmania infantum* antibody levels, expressed as median (interquartile range), at diagnosis (Day 0) and during anti-*Leishmania* treatment (Days 7, 14, 21, and 29) in 15 dogs with clinical leishmaniosis classified as stage C (clinically sick) according to the CLWG classification, as measured by an in-house two-fold serial dilution ELISA

Similarly, Two-fold serial dilution values showed an overall downward trend, with median (IQR) values of day 0 (344,044; 170,602), day 7 (359,103; 199,927), day 14 (325,689; 165,035), day 21 (282,124; 161,585), and day 29 (239,619; 150,714) (Fig. 2). In contrast to single-dilution ELISA, the IQRs were proportionally large (approximately 50–65% of the median), reflecting substantial inter-individual variability in antibody titers. Despite this heterogeneity, the decline in median values from day 7 onward was statistically significant (*p* < 0.001), with pairwise differences detected between day 0 and day 29 (*p* < 0.001), day 7 and day 21 (*p* = 0.047), day 7 and day 29 (*p* < 0.001), and day 14 and day 29 (*p* = 0.0004), supporting a consistent reduction in antibody levels at the group level (Table 2).

**Table 2:** Comparison between weeks. When the Friedman test shows *P* < 0.05, statistically significant differences are shown in the pairwise comparison

Parameter	Friedman test *P* value	Pairwise comparison *P* value
*Clinical Biochemistry*		
Glucose	0.655	
TP	0.823	
BUN	0.181	
CREA	0.921	
Ca	0.935	
P	0.284	
ALT	0.178	
AST	0.658	
ALP	0.392	
GGT	0.161	
TBIL	0.409	
AMY	0.275	
CRP	0.042	Day 0 –Day 29 (*p* = 0.035)
*CBC*		
Red blood cells	0.183	
Hematocrit	0.224	
Hemoglobin	0.199	
MVC	0.384	
MCH	0.617	
MCHC	0.379	
RDW	0.159	
% RETI	0.711	
RETIC	0.86	
RET-HE	0.104	
Leucocytes	0.686	
Neutrophils	0.671	
Lymphocytes	0.053	
Monocytes	0.626	
Eosinophils	0.006	
Basophils	0.494	
Platelets	0.559	
MVP	0.527	
PDW	0.836	
*Serum Protein Electrophoresis*		
TP (g/dl)	0.823	
Albumin (%)	0.001	Day 0 – Day 21 (*p* = 0.002)
		Day 0 – Day 29 (*p* = 0.000)
Albumin (g/dl)	0.021	Day 0 – Day 29 (*p* = 0.037)
Alfa 1 (%)	0.831	
Alfa 1 (g/dl)	0.854	
Alfa 2 (%)	0.057	
Alfa 2 (g/dl)	0.793	
Beta (%)	0.211	
Beta (g/dl)	0.318	
Gamma (%)	0.001	Day 0 – Day 29 (*p* = 0.003)
		Day 7 – Day 29 (*p* = 0.002)
Gamma (g/dl)	0.001	Day 0 – Day 29 (*p* = 0.001)
		Day 7 – Day 29 (*p* = 0.015)
Albumin: Globulin	0.001	Day 0 – Day 21 (*p* = 0.004)
		Day 0 – Day 29 (*p* = 0.000)
*Serology*		
Single-Dilution Elisa (EU)	0.000	Day 0 –Day 21 (*p* = 0.005)
		Day 0 – Day 29 (*p* = 0.000)
		Day 7 – Day 21 (*p* = 0.009)
		Day 7 – Day 29 (*p* = 0.000)
Two-fold serial dilution (EU)	0.000	Day 0 – Day 29 (*p* = 0.000)
		Day 7 – Day 21 (*p* = 0.047)
		Day 7 – Day 29 (*p* = 0.000)
		Day 14 – Day 29 (*p* = 0.0004)
CIC	0.385	

### CIC

Circulating immune complexes level remained broadly stable over the study period (Table 1), with only minor fluctuations in the median (IQR) values across sampling days: day 0 (18.40; 15.70), day 7 (19.30; 14.90), day 14 (17.90; 14.90), day 21 (17.50; 15.00), and day 29 (18.80; 14.00). Overall, these data do not suggest a clear temporal increasing or decreasing trend in CIC during the meglumine antimoniate administration.

From a variability standpoint, the IQR were large relative to the medians at all time points (IQR approximately 74–86% of the median), indicating substantial inter-individual variability in CIC concentrations throughout the study. Despite the stable group-level median, individual dogs likely exhibited heterogeneous CIC values. Consistent with these findings, CIC did not show statistically significant changes over time (*p* = 0.385) (Table 2).

### Laboratory abnormalities detected in CBC and clinical biochemistry

The results of laboratory findings at the time of diagnosis and during the follow-up associated to meglumine antimoniate administration are summarized in Table 1. No statistical significant differences were detected during ongoing meglumine antimoniate plus allopurinol therapy for the CBC the biochemical parameters included in the study: BUN, CREA, Ca, P, ALT, AST, ALP, GGT, TBIL and AMY (Table 2).

### Serum protein agarose gel electrophoresis and CRP

On AGE electrophoretograms, different protein patterns were identified at diagnosis, with polyclonal gammopathy being the most frequent finding (*n* = 10), followed by polyclonal gammopathy associated with an increased alpha-2 globulin peak (*n* = 4), and polyclonal betapathy (*n* = 1). Overall, serum protein electrophoresis showed a progressive improvement in the protein profile over time (Table 1). Albumin exhibited a consistent increase from Day 0 to Day 29, accompanied by a steady rise in the albumin: globulin ratio, indicating a gradual normalization of dysproteinemia. Conversely, the gamma fraction showed a clear decreasing trend from Day 7 onward, consistent with a reduction in hypergammaglobulinemia. Alpha-2 fractions tended to increase slightly over time, although these changes were not statistically significant, whereas alpha-1 and beta fractions remained relatively stable. Total protein concentrations did not display a marked temporal trend; however, widening interquartile ranges during follow-up suggested increasing inter-individual variability. Variability was particularly pronounced at baseline for the gamma and beta fractions, reflecting marked heterogeneity among dogs at diagnosis, with partial convergence of values during follow-up.

AGE electrophoresis also revealed statistically significant changes in some fractions: albumin increased significantly both as a percentage (*p* = 0.001; day 0 vs. day 21 and day 29) and as an absolute concentration (*p* = 0.021; day 0 vs. day 29), while gamma globulins decreased significantly over time (*p* = 0.001), particularly between day 0 or day 7 and day 29. Consequently, the albumin: globulin ratio increased significantly during follow-up (*p* = 0.001), whereas no other protein fractions showed statistically significant temporal changes (Table 2).

Regarding the inflammatory parameter, CRP concentrations were markedly elevated at diagnosis and during the first two weeks of follow-up, followed by a progressive decline from Day 14 to Day 29 (Table 1), suggesting a reduction in systemic inflammation over time, albeit with substantial inter-individual variability, especially at early sampling points. CRP concentrations decreased significantly over the study period (*p* = 0.042), with a significant reduction between Day 0 and Day 29 (*p* = 0.035) (Table 2).

### Co-infections

All dogs included in the study tested negative by the ICT for all evaluated pathogens (*D. immitis*, *E. canis*, and *A. platys*), except for *L. infantum*. Accordingly, all laboratory abnormalities detected at the time of diagnosis were attributed to *L. infantum* infection.

## Discussion

This study demonstrates that completion of meglumine antimoniate therapy at day 28 is associated with significant changes in key serological and inflammatory markers. Specifically, anti-*Leishmania* antibody levels decreased significantly when assessed by both single-dilution and two-fold serial dilution in-house ELISA, accompanied by reductions in CRP and gamma-globulins. Conversely, albumin concentration and the albumin-to-globulin ratio increased significantly after meglumine antimoniate treatment.

In the case of anti-*Leishmania* antibodies, the use of antibody levels to evaluate post-treatment clinical improvement remains a matter of ongoing debate, as highlighted in review articles (Solano-Gallego et al. 2009) and clinical guidelines for canine leishmaniosis (Solano-Gallego et al. 2011), which recommend repeating a quantitative serological test in the same laboratory six months after the initial treatment. However, a very limited number of studies has been focused on evaluate the anti-*Leishmania* antibody kinetics during the meglumine antimoniate administration. The first study to specifically address this issue was published in 1995 (Ferrer et al. 1995), using a Dot-ELISA technique, the authors monitored anti-*Leishmania* antibody titers in 25 dogs with leishmaniosis during treatment with N-methylglucamine and allopurinol, and reported no decrease in titers during the first months after treatment initiation. However, more recent evidence indicates that a decrease in anti-*Leishmania* antibody levels can be detected as early as 30 days after the first meglumine antimoniate injection (Solano-Gallego et al. 2016).

In our study, ELISA technique provides continuous quantitative measurements, as the OD is read objectively by a microplate spectrophotometer. This format enables finer discrimination of incremental changes in antibody levels than titer-based methods, particularly when standard curves, quantitative indices, or serial dilution protocols are used. Although inter-laboratory variability may occur, ELISA performance is generally highly reproducible within a single laboratory when procedures are standardized. In contrast to other serological techniques, such as dot-ELISA (Fisa et al. 1997), an important limitation, particularly when compared with conventional ELISA, relates to the nature of the measurement scale. Dot-ELISA relies on visual assessment of discrete endpoint titers derived from serial serum dilutions. In terms of interpreting the results, dot-ELISA is subjective and depends on the operator’s experience, even when different experienced observers examine the samples. Consequently, it lacks the resolution to detect small or gradual changes in antibody levels, because variations that do not produce a shift to the next dilution step are not reflected in the reported titer; therefore, this technique is not well suited for serological monitoring during ongoing meglumine antimoniate plus allopurinol therapy. In the present study, all samples from each animal were assayed on the same ELISA plate, and all ELISA measurements were performed on the same day, thereby minimizing intra-assay and inter-assay variability.

The results of this study, the kinetics of anti-*Leishmania* antibodies were assessed using an in-house ELISA performed both at a single serum dilution and by two-fold serial dilutions, and significant differences were detected between the time of diagnosis and the end of meglumine antimoniate administration. However, performing two-fold serial dilutions in routine clinical practice would be advisable because, in the absence of strict laboratory conditions that minimize intra-assay and inter-assay variability, testing a single serum dilution may be inappropriate in clinically affected dogs with high anti-*Leishmania* antibody concentrations. In such cases, ELISA signals can become saturated, limiting discrimination among highly positive samples. By contrast, serial dilution ELISA reduces the effective antibody concentration, helping to avoid signal saturation and thereby improving the dynamic range and interpretability of the assay.

From a clinical perspective, it may be useful to aliquot and freeze additional serum samples at predefined time points to both enable direct comparison of antibody kinetics over time and avoid repeated freeze–thaw cycles, which could compromise antibody integrity. This approach is supported by evidence that canine anti-*Leishmania* IgG remains highly stable in serum stored at − 20 °C–− 80 °C for extended periods (6 months, 1 year, 1.5, and 2.5 years) (Marteles et al. 2025). This is particularly important because serological results are most comparable when the same methodology is used and all testing is performed in the same laboratory.

Our results demonstrated that the humoral immune response, as reflected by anti-*Leishmania* antibody production, is highly variable among clinically affected dogs. Notably, although all dogs were classified as sick at diagnosis according to the CLWG criteria, two-fold serial dilution revealed a wide and heterogeneous range of antibody titers, whereas single-dilution testing tended to categorize these dogs uniformly as highly positive, in agreement with other study (Solano-Gallego et al. 2016).

Acute phase proteins (APPs) are serum proteins whose concentrations fluctuate in response to inflammatory stimulation. They are generally categorized as positive APPs, which rise during inflammation including CRP, serum amyloid A, haptoglobin, ceruloplasmin, α2-macroglobulin, α1-acid glycoprotein, fibrinogen, and complement, and negative APPs, which decline such as albumin, transferrin, transthyretin, retinol-binding protein, and adiponectin (Cerón et al. 2005). Several APPs have been used as biomarkers to monitor treatment response in various conditions, including canine leishmaniosis. In dogs with active infection, increased concentrations of CRP and ceruloplasmin have been reported, with CRP showing a more marked increase than ceruloplasmin (Martínez-Subiela et al. 2002). In contrast, CRP concentrations tend to decrease after initiation of anti-*Leishmania* therapy (Daza González et al. 2019a, b a), which is consistent with our findings, as a statistically significant decrease in CRP concentration was detected after completion of meglumine antimoniate administration. Considering our results, albumin, as negative APP, increased significantly after meglumine antimoniate administration and decreased with *L. infantum*–associated inflammation. In this sense, the improvement in clinicopathological abnormalities detected at diagnosis is usually associated with clinical improvement within the first month of therapy in dogs treated with meglumine antimoniate and allopurinol (Manna et al. 2008).

Serum protein electrophoresis is a crucial biochemical technique used for the investigation of a normal distribution of serum protein fractions (albumin, alfa-1, alfa-2, beta and gamma fraction). In small animal veterinary medicine, different serum protein electrophoresis patterns could be detected, from normal pattern to acute-phase protein responses, polyclonal gammopathies, oligoclonal gammopathies or also called restricted polyclonal gammopathies and finally monoclonal or paraproteinemias (Moore and Avery 2019).

This technique involves quantifying total serum protein and then separating proteins into electrophoretic fractions. The main platforms include cellulose acetate electrophoresis (CAE), agarose gel electrophoresis (AGE), and capillary zone electrophoresis (CZE) (Moore and Avery 2019). In current practice, AGE and CZE are preferred because CAE provides lower resolution and sensitivity, offers less reliable densitometric quantification, and may result in overlap of fractions, particularly in the β–γ region. Consequently, CAE is now rarely used and has largely been replaced by other serum protein electrophoresis methods.

In sick dogs, serum protein analysis frequently demonstrates electrophoretic abnormalities (Paltrinieri et al. 2016). These changes are often among the earliest laboratory alterations observed during disease progression and have been reported to correlate positively with clinical severity (Proverbio et al. 2014). The typical electrophoretic profile is characterized by hypoalbuminemia with increased alpha-2 and gamma-globulin fractions, reflecting high circulating antibody and/or autoantibody levels; increases in the beta-globulin region may also be observed in some cases (Meléndez-Lazo et al. 2018). In dogs with clinical *L. infantum* infection, several gammopathy patterns have been described, most commonly polyclonal gammopathy, followed by oligoclonal and biclonal patterns, with monoclonal patterns rarely reported (Paltrinieri et al. 2016). Based on our findings, 28 days of meglumine antimoniate administered in combination with allopurinol was associated with a significant decrease in gammaglobulins, together with normalization of albumin concentration and the albumin-to-globulin ratio. These findings support serum protein electrophoresis as a valuable tool for the diagnosis of canine leishmaniosis and for monitoring patients during meglumine antimoniate treatment and throughout long-term follow-up.

Few studies have evaluated CIC under clinical conditions in CanL. CIC quantification has recently been clinically validated as a diagnostic biomarker for this infection, and its kinetics in response to anti-*Leishmania* treatment have been investigated (Sarquis et al. 2024). In that longitudinal cohort, CIC levels declined after treatment initiation and remained relatively stable throughout the 360-day follow-up, with statistically significant differences detected only between baseline (day 0) and the final visit (day 360). Similarly, in our study, CIC concentrations did not differ significantly across intermediate follow-up time points between day 0 and day 29.

A key limitation of the present study is the small cohort size, which reduces statistical power, amplifies the influence of inter-individual variability, and limits the generalizability of the findings to the broader canine population. This constraint is not unique to our work and has also been reported in other prospective treatment follow-up studies in CanL , including investigations enrolling dogs treated with meglumine antimoniate and monitored at multiple time points (Daza González et al. 2019a, b a) and clinical trials evaluating hepatic (Ikeda-García et al. 2007) and renal biomarkers in treated dogs (Daza González et al. 2019a, b b). Despite this limitation, several design features strengthen the validity of our results: the intensive, high-frequency follow-up enabled a fine-grained temporal characterization of disease evolution and treatment effects, increasing sensitivity to short-term biological changes; and, even with a limited sample size, statistically significant within-dog differences over time were identified. Moreover, the concurrent assessment of a broad panel of biochemical, hematological, and immunological variables provides an internally consistent appraisal of systemic and immune responses, supporting the clinical relevance and robustness of the observed trends despite the restricted cohort size.

In conclusion, this study shows that 28 days of meglumine antimoniate plus allopurinol is associated with early clinical improvement and significant changes in selected biomarkers, supporting the use of serology, serum protein electrophoresis and CRP to monitor early therapeutic response in dogs with clinical leishmaniosis.

## Data Availability

The data that support the findings of this study are available from the corresponding author upon request.
